# Comparative localization of serotonin-like immunoreactive cells in Thaliacea informs tunicate phylogeny

**DOI:** 10.1186/s12983-016-0177-6

**Published:** 2016-09-29

**Authors:** Alberto Valero-Gracia, Rita Marino, Fabio Crocetta, Valeria Nittoli, Stefano Tiozzo, Paolo Sordino

**Affiliations:** 1 Biology and Evolution of Marine Organisms, Stazione Zoologica Anton Dohrn, Villa Comunale, 80121 Naples, Italy; 2 Institute of Marine Biological Resources and Inland Waters, Hellenic Centre for Marine Research, GR-19013 Anavyssos, Greece; 3 Observatoire Océanographique, CNRS, Sorbonne Universités, UPMC Univ Paris 06, Laboratoire de Biologie du Développement de Villefranche-sur-mer, 06230 Villefranche-sur-Mer, France

**Keywords:** Comparative neuroanatomy, Evolution, Immunohistochemistry, Thaliaceans, Tunicata, Zooplankton

## Abstract

**Background:**

Thaliaceans is one of the understudied classes of the phylum Tunicata. In particular, their phylogenetic relationships remain an issue of debate. The overall pattern of serotonin (5-HT) distribution is an excellent biochemical trait to interpret internal relationships at order level. In the experiments reported here we compared serotonin-like immunoreactivity at different life cycle stages of two salpid, one doliolid, and one pyrosomatid species. This multi-species comparison provides new neuroanatomical data for better resolving the phylogeny of the class Thaliacea.

**Results:**

Adults of all four examined thaliacean species exhibited serotonin-like immunoreactivity in neuronal and non-neuronal cell types, whose anatomical position with respect to the nervous system is consistently identifiable due to α-tubulin immunoreactivity. The results indicate an extensive pattern that is consistent with the presence of serotonin in cell bodies of variable morphology and position, with some variation within and among orders. Serotonin-like immunoreactivity was not found in immature forms such as blastozooids (Salpida), tadpole larvae (Doliolida) and young zooids (Pyrosomatida).

**Conclusions:**

Comparative anatomy of serotonin-like immunoreactivity in all three thaliacean clades has not been reported previously. These results are discussed with regard to studies of serotonin-like immunoreactivity in adult ascidians. Lack of serotonin-like immunoreactivity in the endostyle of Salpida and Doliolida compared to *Pyrosomella verticillata* might be the result of secondary loss of serotonin control over ciliary beating and mucus secretion. These data, when combined with other plesiomorphic characters, support the hypothesis that Pyrosomatida is basal to these clades within Phlebobranchiata and that Salpida and Doliolida constitute sister-groups.

**Electronic supplementary material:**

The online version of this article (doi:10.1186/s12983-016-0177-6) contains supplementary material, which is available to authorized users.

## Background

Thaliacea is a class of pelagic tunicates that undergo alternation of generations between the sexual blastozooid stage and the asexual oozooid stage (reviewed in [1]). This clade comprises three orders: Pyrosomatida, Salpida, and Doliolida [2]. Despite a rich literature describing the anatomical characters of thaliaceans, the phylogenetic position within orders is still disputed. Most authors proposed a nested position of Thaliacea within the class ‘Ascidiacea’, thus recognized as a paraphyletic group formed by the Stolidobranchiata, Aplousobranchiata and Phlebobranchiata clades [3–6]. This view suggests that the thaliaceans, with their planktonic life style, diverged from a benthic ancestor. However, there is no consensus on the relationships among Thaliacean orders. Some authors proposed that Pyrosomatida and Salpida group independently from Doliolida [7–9], while more recent works suggested that Pyrosomatida branched off first, and that Salpida and Doliolida are sister groups [10–12]. To date, molecular phylogenetic analyses based on ribosomal markers have been hindered by long-branch attraction [6]. Analyses based on morphological characters have not overcome such error, mainly because of the lack of a more comprehensive taxon sampling, particularly covering all three thaliacean orders [11, 12].

When molecular and morphological phylogenies conflict, neuroarchitectural traits offer a wealth of hitherto largely-unexploited characters which can make valuable contributions to phylogenetic inference even among distantly related groups (e.g., tardigrades, onychophorans, kinorhynchs and priapulids) [13–17]. However, when adopting neural characters, extensive sampling of crown-group representatives is required to assess the origin of evolutionary traits. In thaliaceans, comparative anatomy is particularly problematic due to the complexity of their life cycles and the difficulty of comparing homologous structures. As a consequence, it is essential to sample taxa across all orders.

To better understand thaliacean phylogenetic relationships, we analysed the distribution of serotonin-like immunoreactivity in specimens from the three orders and at different stages of their life cycle. Monoamine serotonin is an ancient and conserved neurotransmitter found throughout Opisthokonta [18]. Serotonin can trigger several physiological functions that range from regulation of ciliary band activity [19], to feeding circadian patterns [20], and influencing emotional state [21]. In addition to neurotransmitter functions, serotonin has also non-neurogenic roles. For instance, it affects cardiac morphogenesis and neural crest cell migration during early mammalian and chicken embryonic development [22–24], modulates gastrulation in echinoderms and insects [25–27], and plays a role in the determination of left-right asymmetry in amphibians and birds [28, 29]. Cellular distribution of serotonin is a reliable biochemical trait to infer phylogenetic hypotheses due to the ancestral nature of this amine, its diffuse role in nervous transmission, and its metabolic and developmental functions [13, 17, 30, 31]. Moreover, the precise classification and description of serotonin-like immunoreactive cells is needed to improve taxonomic comparability [31]. Serotonin-like immunoreactivity in thaliaceans has been described in oozooids of *Doliolum nationalis* (Borgert, 1893) (Doliolida) and *Thalia democratica* (Forsskål in Niebuhr, 1775) (Salpida) [32–34]. Immunoreactivity to serotonin was observed in both species in different organs such as cerebral ganglion, intestine, pericoronal bands, and in a structure termed the ‘placenta’, a single layer of flattened follicle cells that covers the embryo during development [9, 35]. Recently, Braun and Stach classified serotonin-like immunoreactive cells of Ascidiacea, Appendicularia and Thaliacea in three types: one neuronal and two non-neuronal, spherical and elongated respectively. Each of these cell types has a conserved tissue type-specific distribution [34]. However, cell lineage studies are needed to elucidate the origin of serotonin-like immunoreactive cells.

To understand the evolution of the serotonergic system in Thaliacea, three additional species were examined at different successive life cycle stages, including a member of the order Pyrosomatida. Immunohistochemistry against acetylated and tyrosinated α-tubulins was combined with nuclear staining in order to provide overall anatomical landmarks of the nervous system and an antibody against 5HT serotonin was used to describe the distribution of serotonin-like immunoreactive cells. Our study provides a more complete description of thaliacean serotonergic nervous system, with the aim of better understanding the course of neurotransmitter system evolution in this group of invertebrate chordates.

## Results

### Organization of the serotonergic nervous system in the pyrosomatid *Pyrosomella verticillata* (Péron, 1804)

**Fig. 1:**
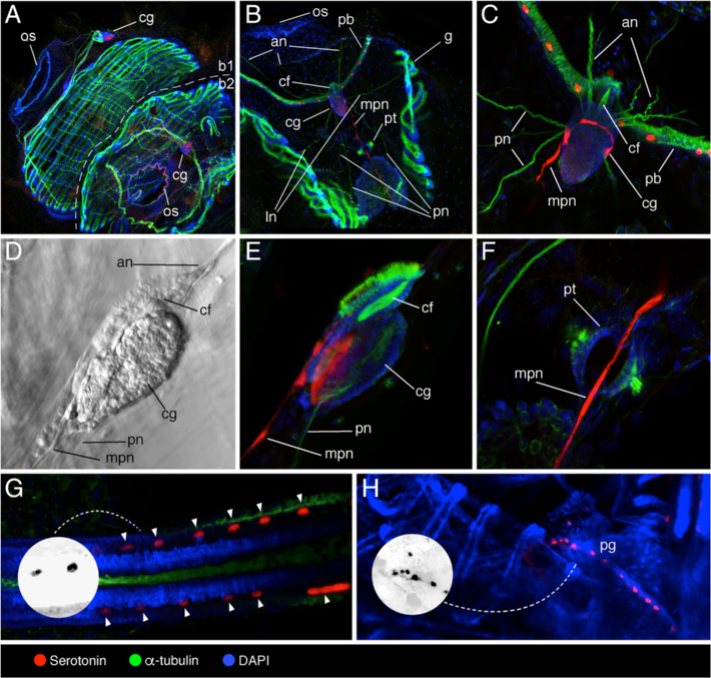
Localization of serotonin-like immunoreactivity, acetylated α-tubulin, and DAPI in *Pyrosomella verticillata* tetrazooid colony. **a** Adult blastozooids (b1 and b2), overview. Oral siphons (os) and cerebral ganglia (cg) highlighted. **b** Mature blastozooid highlighting oral siphon (os), pericoronal bands (pb), ciliated funnel (cf), gills (g), peribranchial tube (pt) and with motor nerves (anterior (an), lateral (ln), posterior (pn) and medial posterior (mpn) nerves) extending from the cerebral ganglion (cg). **c** Detail of the ciliated funnel (cf) and cerebral ganglion (cg) in dorsal view. **d**, **e** Light (**d**) and confocal (**e**) magnification of the cerebral ganglion (cg) (lateral view) in connection with the ciliated funnel (cf). **f** Detail of mpn crossing a peribranchial tube (pt). **g** Detail of the endostyle (serotonin-like immunopositive cells marked with arrowheads), with grayscale invert editing to highlight serotonin-like immunoreactive cell shape (inset). **h** Detail of the posterior part of one adult zooid, highlighting the pyloric gland (pg), with grayscale invert editing to highlight serotonin-like immunoreactive cell shape (inset)

Pyrosomes form tubular colonies consisting of barrel-shaped individual animals (oozooid) that bud off near the posterior closed end of the colony [36]. The nervous system of the pyrosomatid oozooid is an ovoid mass which comprises two regions with contrasting development and function, the neural gland connected to the ciliated funnel, and a voluminous cerebral ganglion [37]. Mature zooids of the tetrazooid colony showed serotonin-like immunoreactivity in neuronal cells of the cerebral ganglion and in the visceral nerve (medial posterior nerve, mpn) running antero-posteriorly and encircling the cerebral ganglion (Fig. 1a–e). The peribranchial tube exhibits two lateral tufts of α-tubulin-positive cilia crossed by the serotonin-like immunoreactive mpn fibres (Fig. 1f). Serotonin-like immunoreactivity was also detected in spherical cell bodies on the pericoronal bands around the oral siphon (Fig. 1c), in two bilaterally symmetrical antero-posterior rows within the endostyle (Fig. 1g), and in a single row in a structure identified as the pyloric gland (Fig. 1h). Early forming and young primary blastozooids growing in the *P. verticillata* tetrazooid colony exhibited axons labelled with the anti-α-tubulin antibody, but no serotonin-like immunosignals were observed (data not shown).

### Organization of serotonergic nervous system in the salpids *Thalia democratica* and *Ihlea punctata* (Forsskål in Niebuhr, 1775)

#### Thalia democratica

**Fig. 2:**
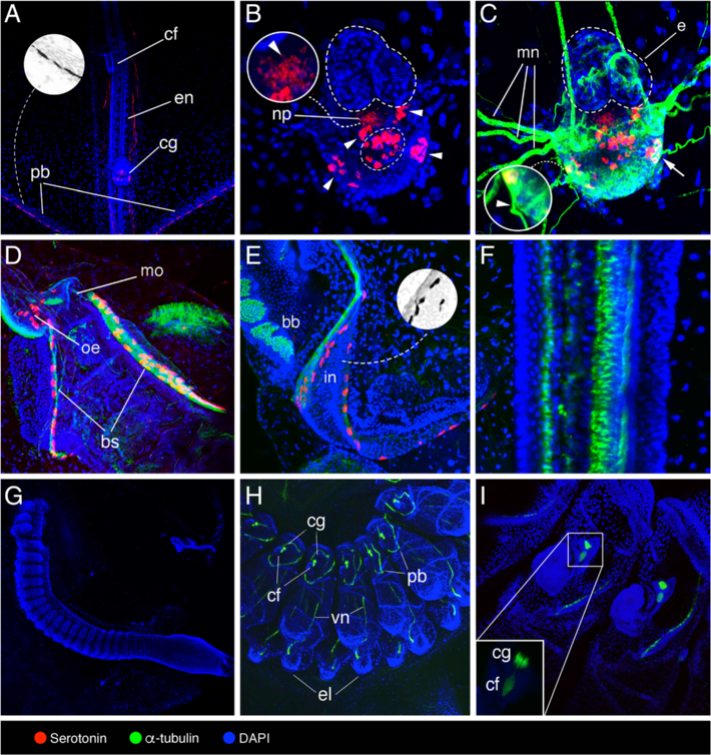
Localization of serotonin-like immunoreactivity, acetylated α-tubulin, and DAPI in *Thalia democratica*. **a**–**f** Adult oozooids. **g**–**i** Aggregate blastozooids. **a** General view of the anterior region that contains the ciliated funnel (cf), endostyle (en), cerebral ganglion (cg), and pericoronal bands (pb), with grayscale invert editing to highlight serotonin-like immunoreactive cell shape in the pericoronal bands (inset). **b** Detail of the cerebral ganglion highlighting peripheral (arrowheads) and central (encircled) serotonin-like immunoreactive cells, and fibres projecting ventrally through the neuropil (arrowhead in the inset). **c** Detail of the cerebral ganglion highlighting eye (e), neuropil (np) (arrow indicates α-tubulin and serotonin co-labelled neuron), and motor nerves (mn) extending from peripheral serotonergic neurons (arrowhead indicates α-tubulin immunoreactive nerve). **d** Detail of mouth (mo), oesophagus (oe) and branchial septum (bs). **e** Magnification of intestine (in) and branchial barrier (bb), with grayscale invert editing to highlight serotonin-like immunoreactive cell shape (inset). **f** Detail of the endostyle. **g** General view of early aggregate blastozooids at developmental stage I sensu Brien [39]. **h**, **i** Details of aggregate blastozooids at developmental stage II sensu Brien [39] highlighting ciliated funnel (cf), cerebral ganglion (cg), pericoronal bands (pb), visceral nerve (vn), and eleoblast (el)

**Fig. 3:**
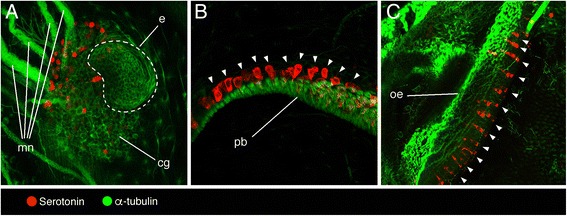
Localization of serotonin-like immunoreactivity and tyrosinated α-tubulin in *Ihlea punctata* oozooids. **a** Cerebral ganglion (cg) with eye (e) and motor nerves (mn) extending from it. **b** Pericoronal bands (pb), serotonin-like immunopositive cells marked with arrowheads. **c** Oesophagus (oe), serotonin-like immunopositive cells marked with arrowheads

A thorough description of the structure of *T. democratica* cerebral ganglion has been provided by Lacalli and Holland [38]. Serotonin-like immunoreactive neurons were found in the posterior half of the cerebral ganglion (Fig. 2a, b, c). A central cluster of serotonin-like immunopositive perikarya was localized near the posterior margin of the neuropil (Fig. 2b). In addition, the cerebral ganglion of *T. democratica* exhibited two paired clusters of serotonin-like immunoreactive neurons laterally (Fig. 2b). Depth color-code analysis of serotonin-like immunoreactivity suggests that a loose bundle of nervous fibres extends ventrally through the neuropil from the central core (Additional file 1). Nervous fibres projecting from the ventral margin of the cerebral ganglion were found to adjoin anteriorly to the optic bundles of the eye (Fig. 2c). Double labelling for serotonin and acetylated α-tubulin suggested that some of the lateral serotonin-like immunoreactive neurons extend fibres as would be expected in case of motor neurons (Fig. 2c). As reported by Pennati et al. [33], serotonin-like immunoreactivity was detected on the pericoronal bands (Fig. 3a), in the digestive system (oesophagus and intestine) (Fig. 2d, e) and on the posterior end of the branchial septum (Fig. 2d). In the first organ, immunoreactive cell bodies have an elongated morphology and are organized in a single row (Fig. 2a), in the second one they are both spherical and elongated and are organized in single and multiple rows (Fig. 2d, e), while in the third one serotonin-like immunoreactive cells are both spherical and elongated, and form two bilateral rows (Fig. 2d). Serotonin-like immunopositive cells were not seen in ciliated funnel (data not shown) and endostyle (Fig. 2f).

Although the anatomy of oozooids and blastozooids of *T. democratica* is similar in many respects, serotonin-like immunoreactivity was not detected in the early aggregate blastozooids derived by strobilation from a posterior stolon of the oozooid (Fig. 2g–i). In blastozooids at developmental stage II sensu Brien [39], labelling of α-tubulins highlighted neural fibres running along the pericoronal bands and in a visceral longitudinal nerve extending to the eleoblast (i.e., a specialized epithelial organ of some thaliaceans) [38] (Fig. 2g–i).

#### Ihlea punctata

In the *I. punctata* oozooids, serotonin-like immunopositive neurons were scattered at the ventral posterior margin of the cerebral ganglion, near the exit of axonal projections extending from it (Fig. 3a). Serotonin-like immunoreactivity was also encountered in spherical cell bodies along the pericoronal bands (Fig. 3b) and in regularly arranged rows in the oesophagus (Fig. 3c). No endostyle was observed in any of the *I. punctata* oozooids examined.

### Organization of the serotonergic nervous system in the doliolid *Doliolina muelleri* (Krohn, 1852)

**Fig. 4:**
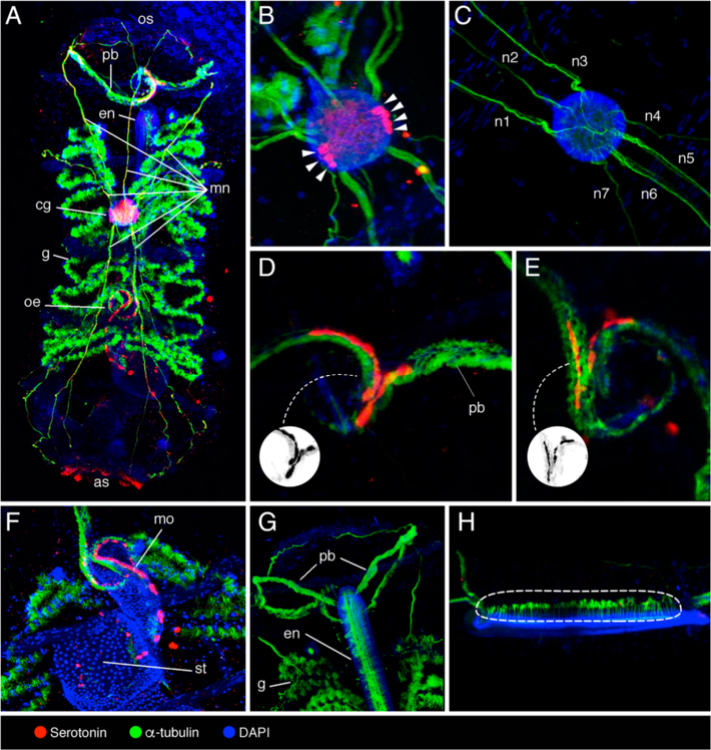
Localization of serotonin-like immunoreactivity, acetylated α-tubulin, and DAPI in *Doliolina muelleri* phorozooid. **a** Dorsal view of the whole mount phorozooid, highlighting oral siphon (os), pericoronal bands (pb), endostyle (en), motor nerves (mn), cerebral ganglion (cg), gills (g), oesophagus (oe), and atrial siphon (as). **b**, **c** Cerebral ganglion with lateral clusters of serotonergic neurons (arrowheads), and motor nerves protruding from it (mn 1–7) at different magnifications. **d**, **e** Pericoronal bands (pb), with grayscale invert editing to highlight serotonin-like immunoreactive cell shape (inset). **f** Initial tract of the digestive system highlighting stomach (st) and serotonergic cells in the mouth (mo). **g** Anterior part of the specimen highlighting pericoronal bands (pb), endostyle (en), and gills (g). **h** Lateral view of the endostyle highlighting the long cilia protruding from it (encircled)

In comparison with salps and pyrosomes, doliolids have a long generation time and their life cycle encompasses different zooids [40]. Their typical body plan is barrel-shaped with two wide siphons and 8–9 circular muscle bands. The neural complex of Doliolida groups the cerebral ganglion (the central nervous system, composed by neurons and the neuropil), the neural gland (an ectodermal structure of unclear function), and the ciliated funnel, sometimes called “vibratile organ” [36]. The cerebral ganglion of *D. muelleri* phorozooids is localized dorsally in the middle of the body, and long nerves emerge from it elongating anteriorly and posteriorly (Fig. 4a). Two clusters of 3–4 serotonin-like immunoreactive neurons are seen laterally in the cerebral ganglion (Fig. 4b), in close proximity to neurons projecting motor nerves (Fig. 4c). A continuous row of serotonin-like immunoreactive spherical cells was seen at the junction of the pericoronal bands (Fig. 4d, e). This region has been previously described as the ciliated funnel in Doliolida [32, 41] but it is probably not homologous to the funnel that links the neural complex (neural gland) to the branchial chamber [42]. Few and sparse spherical and elongated serotonin-like immunoreactive cells were found in the initial tract of the digestive system (mouth and oesophagus) (Fig. 4f). Serotonin-like immunoreactivity was not detected in pericoronal bands and endostyle (Fig. 4g, h).

**Fig. 5:**
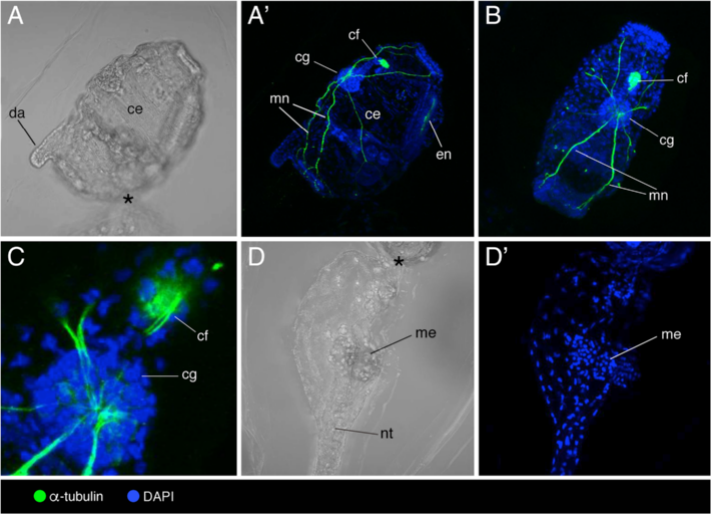
Localization of acetylated α-tubulin and DAPI in tadpole larvae and young zooids of *Doliolina muelleri*. **a**–**d'** Light (**a**, **d**) and confocal (**a**’, **b**, **c**, **d**’) images of a single tadpole larva (**d**, **d**’) connected with a young zooid (**a**, **a**’, **b**, **c**) highlighting cerebral ganglion (cg), ciliated funnel (cf), dorsal appendix (da), endostyle (en), mesoblast (me), major nerves (mn) and notochord (nt); area of contact between zooid and tadpole larvae marked with asterisk (*). **b**, **c** Dorsal view of the young zooid. **d**, **d**’ Detail of the tadpole larva

The barrel-shaped zooid growing in one side of the head of *D. muelleri* tadpole larvae gradually takes on the adult form while the larval tail degenerates (Fig. 5a–c). No serotonin-like immunoreactivity was overall detected in cell bodies or nerves from larvae and young zooids. In young zooids, α-tubulin marked major nerves that appeared to connect a fibre plexus within the neural ganglion to the entire body (Fig. 5a’, b) and a bundle of fibres running through the ciliated funnel (Fig. 5c). No α-tubulin immunoreactivity was observed in tadpole larvae attached to the young zooids (Fig. 5d, d’).

## Discussion

### Serotonin-like immunoreactivity in the nervous system, implications for brain evolution

Based on the localization of serotonin-like immunoreactive neuronal cells with descending projections through the neuropil, Hay-Schmidt [13, 43–47] suggested that an orthogonal organisation of the nervous system was likely present in the last common ancestor of chordates, an idea previously proposed by Garstang [48]. This ancestral condition should be observed also in thaliaceans due to the phylogenetic placement of this clade within ‘ascidians’. We found that serotonin-like immunopositive neurons are symmetrically distributed in the cerebral ganglion of the examined Doliolida and Salpida species and that, at least in *T. democratica*, serotonin-like immunoreactive tracts project transversally through the neuropil (Additional file 1) [34]. Recent works based on the gene expression study of orthologous transcription factors during development suggests that the ascidian CNS holds molecular evidence of brain compartment homology with vertebrate fore-, mid-and hindbrain [3, 49]. In thaliaceans, it will be of great interest to compare gene expression pattern of transcription factors involved in the differentiation of the three organizing centres in the vertebrate brain: the anterior neural ridge, the zona limitans intrathalamica and the isthmic organizer (e.g. *Fgf8*, *Fgf17*, *Fgf18*, *Sfrp1*/*5*, *Hh*, *Wnt1*) [50]. This would help in understanding to which degree the homologous neuroectodermal signalling centers that pattern deuterostome bodies were conserved or diverged in Thaliacea. However, evidence of chordate features in ascidians does appear before metamorphosis, while thaliaceans examined in the present study are all post-metamorphic stages. This suggests that caution is needed when interpreting gene or protein expression patterns in mature forms of thaliaceans.

Based on the expression of several pituitary markers (e.g., *Pitx*, *Pax2*/*5*/*8*, *Six1*/*2*), the ciliated funnel of ascidians has been suggested to be homologous to the adenohypophysis, a major organ of the vertebrate endocrine system that regulates various physiological processes such as stress, growth, and reproduction (reviewed in [51]). In thaliaceans, the ciliated funnel could be responsive to the detection of olfactory information from the environment thus eliciting specific behavioural responses [33]. The evidence presented here concerning the absence of serotonin-like immunoreactive cells in the ciliated funnel of the examined specimens is in agreement with similar reports in appendicularians, ‘ascidians’ and salpids [32, 34, 52–55]. While discounting the use of the serotonergic system in the ciliated funnel of tunicates, this finding suggests that the prominent role played by the local production of serotonin in the pituitary gland is an acquired feature of vertebrates.

### Serotonin-like immunoreactivity in non-neural tissues

The tunicate endostyle, a structure homologous of the vertebrate thyroid, is a ventral U-shaped organ made by folds of the pharyngeal epithelium that secretes mucus for filter feeding [56]. Each mirror-image side of the tunicate endostyle displays between five and nine zones of distinctive cells, including supporting and glandular zones as well as zones with iodinating capacity [57, 58]. In stolidobranch, aplousobranch and phlebobranch ascidians, serotonergic cells were exclusively found in the lateral portion of the endostyle, between zone seven (known to have iodinating capacity), and eight (which consists of ciliated cells) [34, 54, 55, 59–61]. Based on our analysis, serotonin-like immunoreactivity in the endostyle of thaliaceans was detected only in *P. verticillata*, in a lateral zone near a band of ciliated cells, just as in ascidians. The observation that salpids and doliolids lack serotonin-like immunoreactivity in the endostyle provides support for an evolutionary scenario in which Pyrosomatida is the first group branching from the class Thaliacea [11]. However, the absence of serotonin-like immunoreactivity in the endostyle of salpids and doliolids could be a character associated with independent changes in the control of thyroid hormone production rather than an ancestral state; however, this has not yet been verified.

The peripharyngeal (pericoronal) bands of pyrosomatids, salpids and doliolids are rich in ciliated cells and could have a role in mechanoreception [62]. The presence of serotonin-like immunoreactive spherical cells in the pericoronal bands, of ascidians as are in thaliaceans suggests a phylogenetic link between these two tunicate classes [34, 54, 55, 59–61].

The post-pharyngeal digestive tract of tunicates consists of mouth, oesophagus, stomach, intestine, and anus [63, 64]. Digestive functions are also ascribed to the pyloric gland, an organ that begins at the globular gland that encrusts and opens to the intestine. The tunicate pyloric gland is composed of tubules and ampullae that grow from the outer wall of the stomach and is considered to be one of the major synapomorphies of the group [65, 66]. In ascidians, the occurrence of spherical and elongated cell bodies that are serotonin-like immunoreactive is reported in distinct tracts of the digestive system, including oesophagus, stomach and intestine [32, 34, 52, 54, 55]. In our work, serotonin-like immunoreactive cell bodies in the oesophagus of Salpida and Doliolida species, as seen in ascidians, likely reflect a plesiomorphic condition. Conversely, serotonin-like immunoreactivity in the pyloric gland of *P. verticillata* seems to be an independently derived character.

### The serotonergic system is not required in immature forms

We did not detect serotonin-like immunoreactivity in *Thalia democratica* juveniles (blastozooids), in larvae and young asexual zooids of doliolid or in sexual hermaphrodite blastozooid stages of the pyrosomatid examined. The lack of expression of 5HT suggests that serotonin acquires its functionality only in mature thaliacean zooids, thus not having a role in early development. However, serotonin expression may be still present but not detectable with our methods due to low levels or poor permeabilization, prompting for transcriptional activity studies of genes belonging to the serotonin biosynthetic pathway.

### Phylogenetic relationships within Thaliacea

Due to their classification as chordates, the subphylum Tunicata has been central to discussions on the evolution of deuterostomes and craniates [66–76]. Nonetheless, the internal phylogenetic relationships of the tunicate class Thaliacea remain uncertain. Thaliacea is recovered as monophyletic regardless of the number of taxa analysed, the molecular data type used, or the phylogenetic method applied, with the exception of one study which used partial 28S rDNA sequences [10]. Almost all studies agree in grouping Thaliacea as sister group of Phlebobranchiata, one of the classical ‘Ascidiacea’ groups (where ‘Ascidiacea’ = Phlebobranchiata + Stolidobranchiata + Aplousobranchiata). We assume Phlebobranchiata as out-group for our phylogenetic comparison, due to the placement of this clade as adelphotaxon of Thaliacea in many studies [4, 7, 66, 77].

**Fig. 6:**
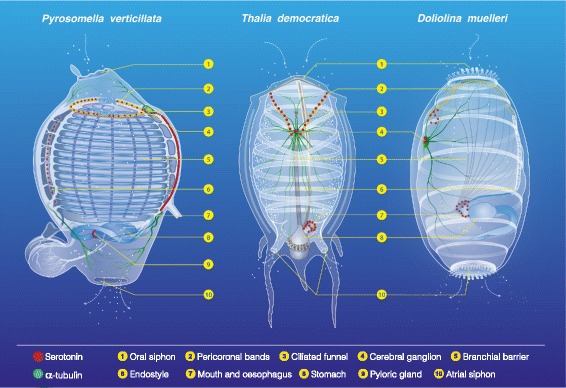
The serotonin-like immunoreactive nervous system in Thaliacea. serotonin-like immunopositive cells in adult *Pyrosomella verticillata*, *Thalia democratica* and *Doliolina muelleri*

A scheme summarizing the differential spatial distribution of serotonin-like immunoreactivity among organs in Thaliacea is shown in Fig. 6.

**Fig. 7:**
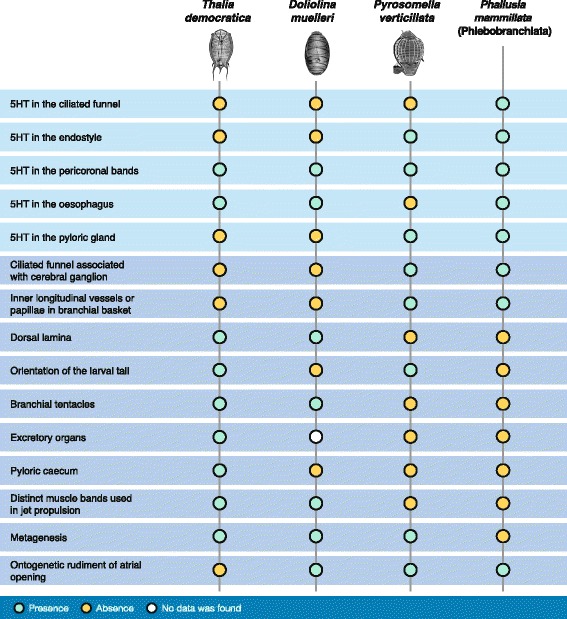
Phylogenetic characters. Comparison of serotonin-like immunoreactivity distribution and selected plesiomorphies in thaliacean orders and in the phlebobranch species, *Phallusia mammillata*. Non-neural data from [35, 52, 61, 76, 77]

Character comparisons suggesting that pyrosomatids originated early in the evolutionary history of thaliaceans include the presence of serotonergic cells in the endostyle and pyloric gland as in the phlebobranch *Phallusia mammillata* [54]. This condition is not present in the Salpida or Doliolida species examined, as discussed above. Since the presence of serotonin-like immunoreactivity alone cannot be considered as an uncontroverted character of phylogenetic value, we supplemented our molecular data with ten morphological and life cycle characters extracted from the literature [32]. Apomorphies such as the existence of inner longitudinal vessels in branchial basket and the presence of ontogenetic rudiment of atrial opening are common features shared just between Pyrosomatida and Phlebobranchiata [66]. The ciliated funnel is a very variable organ both with respect to its anatomy and its topology. It is associated with the cerebral ganglion in appendicularians, pyrosomatids and ‘ascidians’, but not in salpids nor in doliolids [37, 78–80]. Otherwise, the topology of the ciliated funnel in salpids is distinct from that of pyrosomatids and ‘ascidians’ in that it is not continuous to the pericoronal bands [37, 42]. Further, the presence of dorsal lamina, branchial tentacles and distinct muscle bands used in jet propulsion in Salpida and Doliolida [66] supports a sister group relationship between these two thaliacean orders. By applying a principle of parsimony, these characters (Fig. 7) seem to favour the phylogenetic hypothesis in which Pyrosomatida, an order often classified within the ‘ascidians’ [11, 80, 81], and not Doliolida [6], is the first branching group from Thaliacea.

## Conclusion

Here we present a study of serotonergic immunoreactivity in the three thaliacean orders, and provide a first description of the pyrosomatid serotonergic system. The analysis of the distribution of serotonin-like immunopositive cells in adult thaliacean oozooids appear to depict shared characters with ascidians. Remarkably, serotonin-like immunoreactivity is not present in immature thaliacean zooids, suggesting that this amine is not crucial for the morphogenesis of the species examined. Differences in serotonin-like immunoreactive arrangement in endostyle, initial tract of the digestive system and pyloric gland, plus a review of life cycle and morphological data, prompt us to support the phylogenetic hypothesis in which Pyrosomatida is the first Thaliacean order that diverged from the Ascidiacea clade, thus positioning Salpida and Doliolida as sister groups. Data from more species and the support of molecular based phylogenetic and/or phylogenomic analyses will be crucial to make more robust the relationships among different clade of Thaliaceans.

## Methods

### Animal collection and identification

Samples were collected in the Western Mediterranean using vertical plankton tows-200 μm mesh size-in the localities of Rade de Villefranche-sur-Mer (France) (43°42′18″N 7°18′45″E) (*Pyrosomella verticillata* and *Ihlea punctata*) and Gulf of Naples (Italy) (40°48′5″N 14°15′E) (*Thalia democratica* and *Doliolina muelleri*). Specimens were identified under stereomicroscope following taxonomic keys in [82] and [83].

### Whole mount immunocytochemistry and imaging

Specimens were fixed in 4 % paraformaldehyde/0.1 M MOPS pH 7.4 containing 0.5 M NaCl, overnight at 4 °C. After several washes in phosphate buffered saline (PBS), samples were treated with 0.5 mg/ml cellulase (Sigma C1184) in PBS pH 5.5 for 10 min at 37 °C in order to partially digest the tunic and facilitate antibody penetration. Following this, incubations were carried out on a rotating shaker. Specimens were permeabilized for 20 min in PBS/0.25 % Triton X-100 (PBTr). A blocking step was performed with 30 % heat-inactivated Normal Goat Serum (NGS) in PBTr for 2 h, prior to incubating specimens with primary antibodies-anti-5HT (serotonin) (Immunostar 20080), anti-tyrosinated α-tubulin (Sigma T9028), and anti-acetylated α-tubulin (Sigma T6793)-diluted 1:300 in PBS containing 0.1 % Tween-20 (PBST) and 30 % NGS, for 60 h at 4 °C. After extensive washes in PBST, samples were incubated at 4 °C overnight with secondary antibodies-goat anti-rabbit IgG-Alexa 488 and goat anti-mouse IgG-Alexa 647-diluted 1:400 in blocking buffer (1 % BSA in PBST). All samples were washed thoroughly in PBS. All specimens except *Ihlea punctata* were counterstained with DAPI (1 μg/ml in PBS) for nuclear labelling. Control experiments were run in parallel by omitting primary antibodies.

Image acquisition was performed on Zeiss LSM 510 Meta and Leica SP5 confocal microscopes. Z-stack images were analyzed and processed with Fiji and Photoshop CS6 (Adobe). Figure plates were made with Illustrator CS6 (Adobe). Brightness/contrast, inversion and colour balance adjustments where applied, were applied to the entirety of the image and not to parts thereof.

## Additional file

Additional file 1: Visual assessment of serotonin-like immunoreactivity in *Thalia democratica* cerebral ganglion. (A–E) Five consecutive frontal sections ranging dorsal to ventral from Z = 14 to Z = 18 every 4.54 μm, showing elongating serotonin-like immunoreactive bundle (squared line). (F) Color-coded 2D image from hyperstacks Z = 14–18, showing different depth distribution of lateral clusters of serotonin-like immunoreactive neurons (arrows), central cluster of serotonin-like immunoreactive neurons (dashed line) and serotonin-like immunoreactive nervous fibre bundle (squared line). (JPG 1000 kb)
