# Supplementary figures and images for: Comparative localization of serotonin-like immunoreactive cells in Thaliacea informs tunicate phylogeny
Source: Front Zool. 2016 Sep 29;13:45. doi: 10.1186/s12983-016-0177-6 (PMC5041399; doi:10.1186/s12983-016-0177-6)

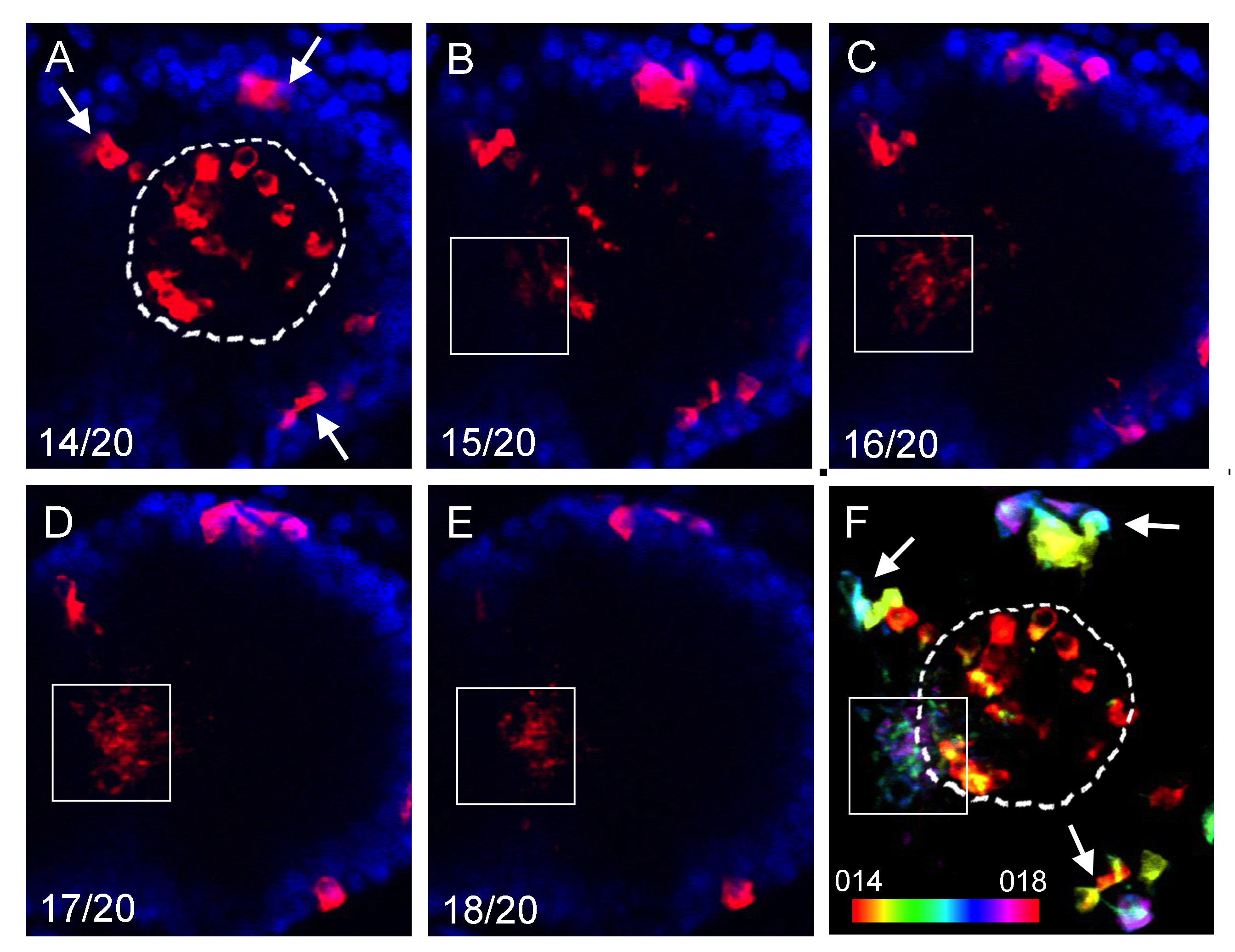

Supplement: Additional file 1: — Visual assessment of serotonin-like immunoreactivity in Thalia democratica cerebral ganglion. (A–E) Five consecutive frontal sections ranging dorsal to ventral from Z = 14 to Z = 18 every 4.54 μm, showing elongating serotonin-like immunoreactive bundle (squared line). (F) Color-coded 2D image from hyperstacks Z = 14–18, showing different depth distribution of lateral clusters of serotonin-like immunoreactive neurons (arrows), central cluster of serotonin-like immunoreactive neurons (dashed line) and serotonin-like immunoreactive nervous fibre bundle (squared line). (JPG 1000 kb) [file 12983_2016_177_MOESM1_ESM.jpg]
